# Epithelial immune activation and intracellular invasion by non-typeable *Haemophilus influenzae*

**DOI:** 10.3389/fcimb.2023.1141798

**Published:** 2023-04-24

**Authors:** Mary A. Brown, Sophie B. Morgan, Gillian E. Donachie, Katie L. Horton, Ian D. Pavord, Carolina V. Arancibia-Cárcamo, Timothy S. C. Hinks

**Affiliations:** ^1^ Respiratory Medicine Unit and National Institute for Health Research Oxford Biomedical Research Centre, Experimental Medicine Division, Nuffield Department of Medicine, John Radcliffe Hospital, University of Oxford, Oxford, United Kingdom; ^2^ School of Clinical and Experimental Sciences, University of Southampton Faculty of Medicine, Southampton, United Kingdom; ^3^ Translational Gastroenterology Unit, Nuffield Department of Medicine, Experimental Medicine, University of Oxford, Oxford, United Kingdom

**Keywords:** asthma, *Haemophilus influenzae*, epithelial cell biology, innate immunity, COPD

## Abstract

Type-2 low asthma affects 30-50% of people with severe asthma and includes a phenotype characterized by sputum neutrophilia and resistance to corticosteroids. Airways inflammation in type-2 low asthma or COPD is potentially driven by persistent bacterial colonization of the lower airways by bacteria such as non-encapsulated *Haemophilus influenzae* (NTHi). Although pathogenic in the lower airways, NTHi is a commensal of the upper airways. It is not known to what extent these strains can invade airway epithelial cells, persist intracellularly and activate epithelial cell production of proinflammatory cytokines, and how this differs between the upper and lower airways. We studied NTHi infection of primary human bronchial epithelial cells (PBECs), primary nasal epithelial cells (NECs) and epithelial cell lines from upper and lower airways. NTHi strains differed in propensity for intracellular and paracellular invasion. We found NTHi was internalized within PBECs at 6 h, but live intracellular infection did not persist at 24 h. Confocal microscopy and flow cytometry showed NTHi infected secretory, ciliated and basal PBECs. Infection of PBECs led to induction of CXCL8, interleukin (IL)-1β, IL-6 and TNF. The magnitude of cytokine induction was independent of the degree of intracellular invasion, either by differing strains or by cytochalasin D inhibition of endocytosis, with the exception of the inflammasome-induced mediator IL-1β. NTHi-induced activation of TLR2/4, NOD1/2 and NLR inflammasome pathways was significantly stronger in NECs than in PBECs. These data suggest that NTHi is internalized transiently by airway epithelial cells and has capacity to drive inflammation in airway epithelial cells.

## Highlights

NTHi is internalised by primary bronchial epithelial cells. It activates innate responses including CXCL8, IL-1β, IL-6 and TNF, independently of invasion, with more activation in nasal than bronchial epithelial cells, despite NTHi being a nasal commensal.

## Introduction

Asthma is a complex, heterogeneous condition characterized by airway hyperreactivity and airways inflammation, classically including type 2 immune responses mediated by innate and adaptive T cells, eosinophils and mast cells (Hinks et al., 2015). However, other phenotypes exist and 40-50% of patients treated with high doses of inhaled corticosteroids (ICS) have a normal eosinophil count (Hinks et al, 2021) with 16-20% displaying neutrophilic airways inflammation (Simpson et al., 2006; Schleich et al., 2013), which is poorly responsive to therapeutic corticosteroids, and shows enhanced susceptibility to exacerbations. The mechanisms of neutrophilic asthma are poorly understood, but it has been associated with the presence of the gram-negative bacterium *Haemophilus influenzae (H. influenzae)*, particularly the non-encapsulated or non-typeable *H. influenzae* strains (NTHi) (Brown et al., 2022). NTHi is the most commonly isolated airway pathogen in metagenomic studies of airways diseases (Simpson et al., 2016; Abdel-Aziz et al., 2019) and is linked to exacerbations (Yang et al., 2018; Zhang et al., 2020). NTHi is a commensal organism in the nasopharynx (NP), colonizing the nasopharynx of even most healthy people (Miyamoto and Bakaletz, 1997; Pichichero and Almudevar, 2016), but is an opportunistic pathogen in the lower airways, possibly due to differential activation of mucosal immunity. Several longitudinal studies have demonstrated that NTHi is capable of persisting within the respiratory tracts of patients with airways diseases for years at a time (Murphy et al., 2004; Pettigrew et al., 2018). Some data suggest NTHi is capable of being internalized into airway epithelial cells, although it is unclear whether the bacteria can replicate and persist within the epithelial monolayer (Morey et al., 2011; Baddal et al., 2015). NTHi may contribute to inflammatory signaling and type-2 low airway inflammation associated with asthma, and is a significant driver of cytokine production in the lung, whilst elimination of NTHi using long-term azithromycin treatment was associated with reduced concentrations of IL-1β and IL-6 in sputum (Shukla et al., 2021).

It is not understood how NTHi persists within the human airways, or how this bacterium is tolerated as an innocuous commensal in the upper respiratory tract, whilst acting as a pathogen in the lower airways contributing to steroid-resistant neutrophilic asthma. We sought to determine whether NTHI is internalized into human airway epithelial cells, to determine its capacity to persist within these cells and to evoke neutrophilic inflammation, and to compare these effects in epithelial cells from the upper and lower airways.

## Methods

### Cell culture

The Calu-3 lung adenocarcinoma cell line, commonly used as a model for bacterial infection and immune activation (Sajjan et al., 2008; Hirakata et al., 2010; Stewart et al., 2012), was cultured according to American Type Culture Collection (ATCC) guidelines in Eagles minimum essential media supplemented with FBS and sub-cultured upon reaching 80% confluence. The immortalized BEAS-2B (ATCC) bronchial epithelial cell line, widely used for asthma studies (Han et al., 2020), was maintained in collagen-coated flasks in serum-free airway epithelial cell media (PromoCell) supplemented with 100μg/mL Penicillin/Streptomycin (Gibco). To sub-culture, trypsin–EDTA was supplemented with 0.5% polyvinylpyrrolidone, and soybean trypsin inhibitor (0.5 mg/mL in PBS) was used to inactivate the trypsin at a 1:1 ratio.

Primary bronchial epithelial cells (PBECs) were obtained from right and left lower and middle lobe 3^rd^ to 5^th^ order bronchi at bronchoscopies performed on healthy control donors in accordance with the British Thoracic Society guidelines (2014) and established research protocols (Jarjour et al., 1998; British Thoracic Society, 2014), after written informed consent. The study was reviewed by Oxford Research Ethics Committee B (18/SC/0361) and Leicestershire, Nottinghamshire and Rutland Ethics Committee, UK (08/H0406/189). Nasal epithelial cells (NECs) were collected from healthy volunteers using cytology brushes. Samples were washed in phosphate buffered saline (PBS), centrifuged at 200 x g for 10 min, resuspended in serum-free media (PromoCell) and plated at 5000-8000 cells/cm^2^ in 6-well plates pre-coated with PureCol (Advanced BioMatrix) for initial culture. Primary nasal and bronchial epithelial cells were seeded for maintenance at 4000-8000 cells/cm^2^ in T75 flasks pre-coated with PureCol in serum-free airway epithelial cell media and sub-cultured at 70-80% confluence. To sub-culture, trypsin–EDTA was supplemented with 0.5% polyvinylpyrrolidone, and soybean trypsin inhibitor (0.5 mg/mL in PBS) was used to inactivate the trypsin at a 1:1 ratio.

### Air-liquid interface culture

NECs or PBECs were cultured on PureCol-coated 0.4 μm pore polyester membrane permeable inserts (Corning) in serum-free airway epithelial cell media, brought to air-liquid interface (ALI), replacing basal media with ALI media (StemCell Pneumacult). At ALI, media were exchanged every 2 days and apical surfaces were washed with PBS weekly to disperse accumulated mucus for a minimum of four weeks.

### NTHi stock culture

NTHi strains were grown overnight on chocolate agar at 37°C, 5% CO_2_, then inoculated in Brain-Heart Infusion supplemented with 2 μg/mL NAD and 10 μg/mL hemin and incubated in a shaking incubator at 200 x g until reaching OD_600_ of 0.4 to 0.8. A list of NTHi strains and relevant citations is included in **Supplemental Figure 8** (Fox et al., 2008; Martí-Lliteras et al., 2011). Clinical strains were a generous gift from the existing collection of Dr. Derek Hood at MRC Harwell, whilst lab strain NCTC11931 was bought from the National Tissue Culture Collection.

### Bacterial internalization assay

Confluent submerged or ALI cultures were inoculated with NTHi at multiplicity of infection (MOI) 5-25 for varying durations at 37°C in humidified 5% CO_2_. Extracellular bacteria were killed with 200 μg/mL gentamicin for 1.5 hours, and cells washed twice with PBS, as previously described in the literature (Valour et al., 2013). Cells were lysed using 1% Triton-X 100 for 10-15 min to release intracellular bacteria, then plated in 10-fold dilutions on chocolate agar to estimate bacterial colony forming units (CFU) (Sharma and Puhar, 2019). Paracellular and adherent bacteria were estimated by trypsinising cells to expose paracellular bacteria to gentamicin, or by eliminating the gentamicin step to include adherent bacteria.

### Immunofluorescence imaging of ALI cultures

PBEC cultures grown at ALI for a minimum of 28 days were inoculated with CellTrace™ CFSE (Invitrogen)-labelled NTHi strain 398. Transwells were fixed in 4% paraformaldehyde for 15 min, washed with PBS, incubated with blocking buffer for 1 h then with primary antibodies overnight and secondary antibodies for 1-2 h in 1% BSA and 0.3% Triton-X100 at 4°C, before mounting onto glass and cured for 24 h prior to imaging.

### Flow cytometric sorting of ALI cells

Mature ALI cultures of NECs and PBECs infected for 1 h with CellTrace™ Far Red-labelled NTHi strain 398 at MOI of 50 were treated with gentamicin, stained with Tubulin Tracker™ (ciliated cells), then treated with 0.1 mM trypsin, 0.9 mM EDTA for 15 minutes to dissociate the monolayer. The single-cell suspension was then stained with markers for basal cells (CD49f), secretory cells (CD66C) and Zombie™ Near IR live/dead stain, and sorted on a BD FACS Aria™ III (Lepanto et al., 2014; Bonser et al., 2021).

### qPCR and PCR array

Whole-cell RNA was extracted using the Qiagen RNEasy™ kit and for conventional qPCR reverse-transcribed using Applied Biosystems High-Capacity cDNA reverse transcription kit and qPCR performed with SYBR Green. Primers listed in **Supplemental Figure 9**. For Fluidigm DeltaGene™ assays, RNA was converted using proprietary Fluidigm™ cDNA reverse-transcription kit before chip-based Fluidigm Biomark™ PCR array. Data were analyzed using the delta-delta Ct method (Livak and Schmittgen, 2001). Experiments have used both RPL13A and GAPDH housekeeping genes, yielding comparable results.

### Statistical analysis

Statistical analyses were performed using Prism (GraphPad Software, 2021) and used a combination of parametric tests and non-parametric tests (Choi, 2016). Log-normally distributed data were log transformed, after addition of a small value if data contained zero values. For parametric data, groups were compared with Students t-tests (two groups) or analysis of variance (ANOVA)(multiple groups). One-way ANOVAs were used to compare a single independent variable, and two-way ANOVA where there were two independent variables, followed by a Sidaks multiple comparison test to evaluate the difference between all groups.

## Results

**Figure 1:**
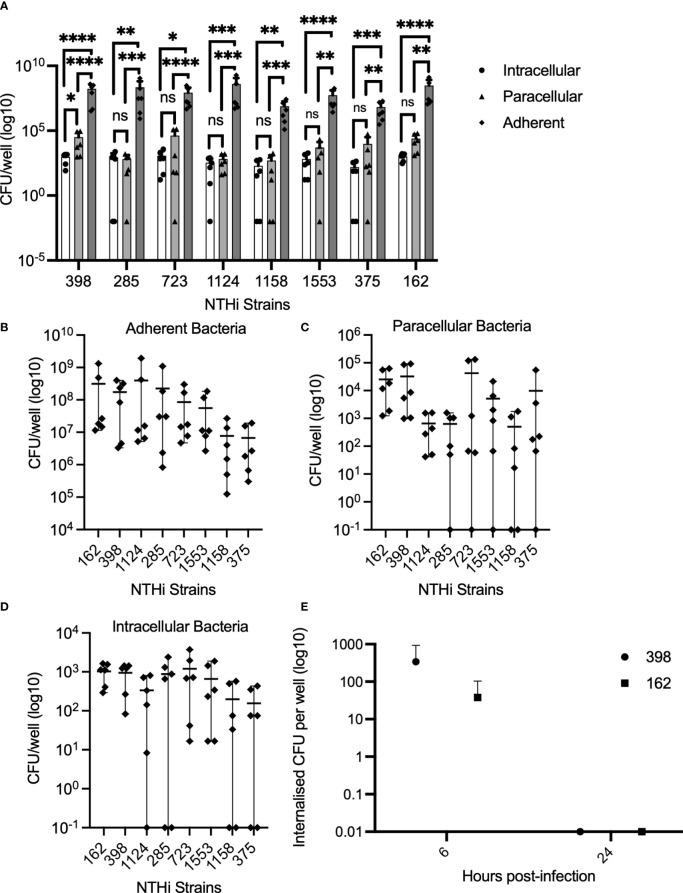
NTHi is temporarily internalized within the epithelial monolayer, and can invade intracellularly and paracellularly. Enumeration of bacterial adhesion and internalization into Calu-3 cells and the paracellular space following incubation with NTHi strains for 4 hours was performed. n=3 independent replicates, with dots representing the mean of technical duplicates for each independent experiment **(A–D)**. **(A)** Adherent bacteria account for >99% of bacteria interacting with epithelial cells. By 2-way ANOVA, there was overall a significant difference between number of intracellular bacteria and adherent bacteria, and between paracellular and adherent bacteria numbers by 2-way ANOVA (p<0.0001). Results from Sidaks *post-hoc* test is indicated for individual strains. **(B)** Numbers of adherent bacteria varied significantly between strains. Overall difference between strains by 2-way ANOVA, p<0.0001. **(C)** Numbers of paracellular bacteria did not differ significantly between strains by 2-way ANOVA. **(D)** Numbers of internalized bacteria varied significantly between strains. Difference between strains by 2-way ANOVA, p=0.0003. **(E)** NTHi does not persist within PBECs in ALI culture in the absence of extracellular bacteria. Viable internalized bacteria of strains 398 and 162 were detectable at 6 hours by culture of lysed cells, but not at 24 hours post-infection in cells grown at ALI and maintained in media containing gentamicin to eliminate replication by extracellular bacteria in the media. Dots represent means of n=3 biological replicates from healthy control donors. Difference between time points not significant by 2-way ANOVA and *post-hoc* Sidaks multiple comparisons test. Throughout, **** corresponds to P ≤ 0.0001, *** indicates P ≤ 0.001, ** means P ≤ 0.01, * indicates ≤ 0.05, anything P > 0.05 is ns.

To investigate the physical interactions between NTHi and the epithelium, localization of NTHi within the epithelial monolayer was evaluated by using a modified gentamicin exclusion assay. In Calu-3 cells grown to confluence in submerged culture using traditional media with FBS supplementation, adherent bacteria account for over 99% of all bacteria interacting with the epithelial cells (**Figure 1A**). Comparable numbers of NTHi were isolated from the paracellular and intracellular spaces across strains (2-way ANOVA with Tukeys multiple comparisons test p=0.08) (1A). The number of adherent bacteria varied significantly between strains (p<0.0001 by 2-way ANOVA), with strain 1158 and 375 being markedly lower than the most adherent strain, strain 162 (7.7x10^6^ CFU/well and 6.7x10^6^ CFU/well vs 3.1x10^8^ CFU/well, respectively) (**Figure 1B**). Likewise, numbers of internalized bacteria similarly varied between strains (difference between strains by 2-way ANOVA p=0.0003), with lowest numbers observed with 1158 and 375, and highest for strain 162 (1.9x10^2^ CFU/well and 1.6x10^2^ CFU/well vs 1x10^3^ CFU/well, respectively) (**Figure 1D**). Numbers of paracellular bacteria varied, but were not significantly different between strains (**Figure 1C**). In the absence of extracellular bacteria, NTHi does not appear to persist within primary bronchial epithelial monolayers grown in serum-free ALI culture in the presence of gentamicin to eliminate extracellular bacteria (**Figure 1E**). In PBECs grown at ALI in serum-free medium, NTHi strains 398 and 162 were internalized at 6 h post-infection (3.4x10^2^ CFU/well and 3.8x10^1^ CFU/well, respectively), but did not appear to persist in the absence of extracellular bacteria, with culturable internalized bacteria disappearing by 24 h post-infection (**Figure 1E**). Likewise, in PBECs grown in serum-free submerged culture, internalized bacteria were no longer culturable by 24 h post-infection in cells treated with gentamicin to eliminate extracellular bacteria (data not shown). These results suggest that NTHi does not persist within epithelial cells alone. Continuous internalization of bacteria is possible in a model of PBECs grown in submerged culture in antibiotic-free, serum-free medium where extracellular bacteria are present, although intracellular numbers decline over time despite substantial replication of extracellular bacteria in the media (mean media CFU/well of strains 398 and 1607 increasing by 58-fold and 42-fold respectively by 168 hours post-infection) (**Supplemental Figure 1**).

**Figure 2:**
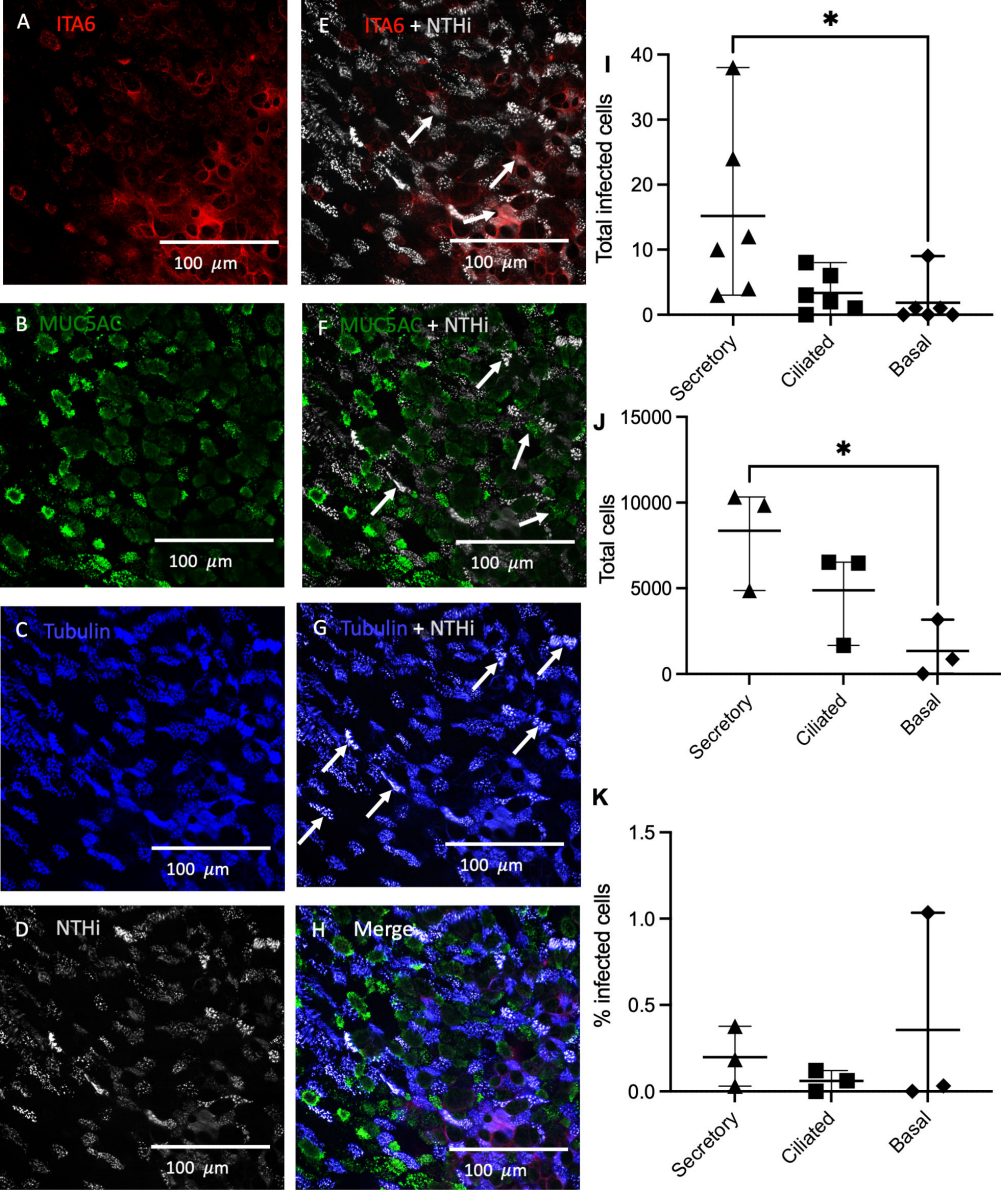
Rates of NTHi internalization in different epithelial cell types. PBECs cultured at ALI were infected with NTHi CFSE-stained strain 398 for 4 hours, fixed in 4% PFA and then stained for ciliated, basal and goblet cell markers. **(A)** Basal cell marker Integrin subunit alpha-6 (ITA6). **(B)** MUC5AC marks secretory cells in green. **(C)** Acetylated alpha-tubulin indicates ciliated cells. **(D)** NTHi are labelled in grey to indicate CFSE-stained bacteria. **(E)** ITA6 and NTHi Merge. **(F)** MUC5AC and NTHi merge. **(G)** Acetylated alpha-tubulin and NTHi merge. **(H)** Merge of all staining. Arrows denote clusters of internalized NTHi. PBECs cultured at ALI and inoculated with NTHi were flow-sorted to determine proportion of each cell type infected by NTHi. **(I)** Internalization of NTHi expressed as total number of infected cells. **(J)** Relative composition of epithelial culture by cell type. **(K)** Percent of cells infected by cell type. n=3 biological replicates from healthy control donors, dots represent means of replicates. Differences compared by 2-way ANOVA with *post hoc* *, P<0.05 for Sidaks multiple comparisons test.

The airway epithelium comprises a variety of distinct cell types (Crystal et al., 2008). To determine which cell types are particularly susceptible to NTHi invasion, and hence a potential reservoir of persistent airways infection *in vivo*, the prevalence of NTHi infection within different types of epithelial cell was characterized in an ALI model of differentiated epithelium using immunofluorescence imaging and flow cytometry (**Figure 2**). Immunofluorescence imaging of PBECs cultured at ALI inoculated with NTHi 398 stained with CFSE demonstrated that NTHi was able to infect all differentiated epithelial types (**Figures 2A–H**). Co-localization with NTHi was most pronounced with ciliated epithelial cells, whilst co-localization was only moderately observed in basal and secretory cells (**Figures 2E–G**). Flow-sorting of mature PBECs cultured at ALI confirmed that all epithelial cell types were permissive to NTHi infection (**Figures 2I–K**, for gating strategy see **Supplemental Figure 2**). The total number of infected cells in each sample was highest for secretory cells (7.2-fold and 3.5-fold higher compared to basal and ciliated cells respectively), while modest numbers of ciliated and basal cells were infected (secretory vs basal cells, p=0.03, one-way ANOVA and *post hoc* Tukeys) (2I). The total number of infected and uninfected cells of each type in the sample was evaluated, demonstrating that the total numbers of each cell type mirrored the numbers of infected cells, with a significantly higher total number of secretory compared to basal cells (ordinary one-way ANOVA with Tukeys multiple comparisons test p=0.035) (2J). Basal cells were most highly infected by percent infection (0.35%), followed by secretory cells (0.18%), with the lowest proportion of infected ciliated cells (0.06%) (2K), but these differences did not reach statistical significance. Thus, all epithelial cell types were permissive to NTHi infection. While secretory cells were infected in the highest numbers, basal cells were proportionally the most highly infected given the lower total numbers of basal cells in the ALI cultures, with ciliated cells being moderately infected.

**Figure 3:**
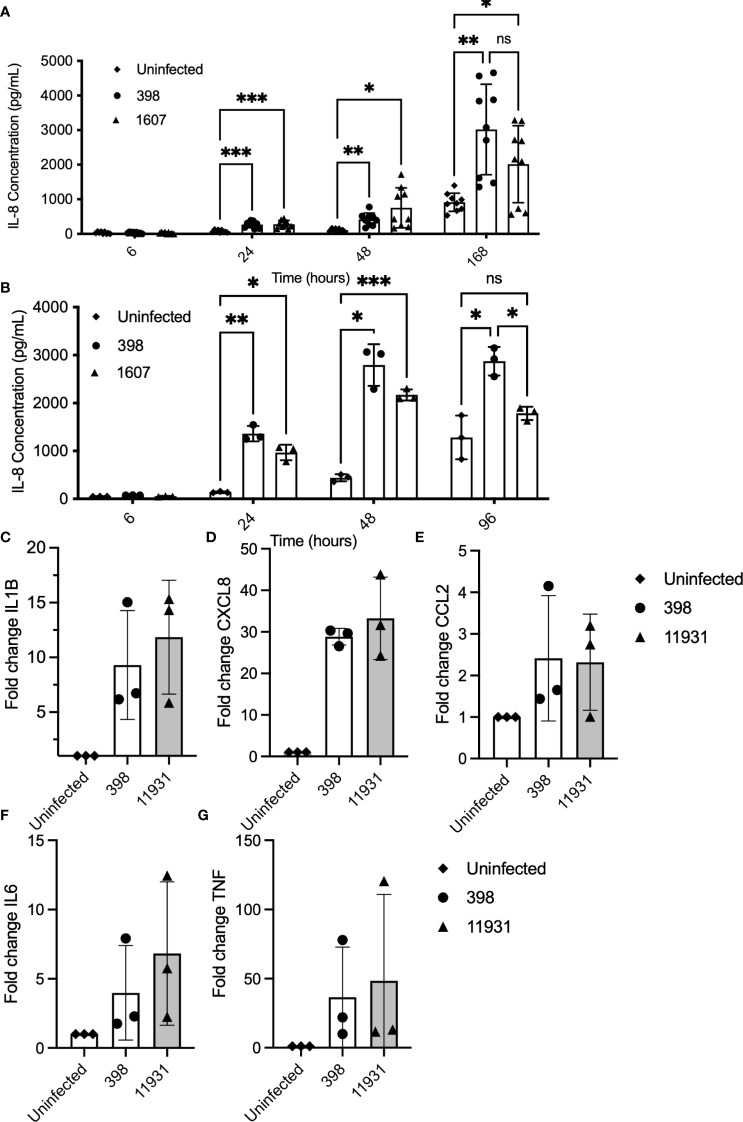
Cytokine induction in response to NTHi infection occurs independently of on internalization rate. PBECs and NECs in serum-free submerged culture were inoculated with a highly internalized strain (398) and less internalized strain (1607). Supernatants were collected at various timepoints and levels of IL-8 (CXCL8) were evaluated using ELISA. **(A)** IL-8 production was significantly induced in cells treated with either strain of NTHi from 24 hours post-infection. IL-8 production did not differ significantly between strains at any time point. Significance was evaluated using 2-way ANOVA with Sidaks multiple comparison tests to evaluate significance between each treatment and timepoint. n=3 biological replicates. **(B)** IL-8 production was elevated in NECs at 24 and 48hpi after exposure to NTHi. Significance was evaluated using 2-way ANOVA with Sidaks multiple comparison tests to evaluate significance between each treatment and timepoint. n=3. **(C–G)**. When evaluated using qPCR, infection with a more highly internalized NTHi strain did not correlate with more robust immune activation in Calu-3 cells inoculated with NTHi strains 398 (high internalization) compared with NTHi lab strain 11931 (low internalization). Each marker was significantly increased in cells treated with NTHi, but there was no significant difference in induction between strains. Significance was evaluated using 2-way ANOVA with Sidaks *post-hoc* multiple comparisons tests to evaluated the difference between strains. n=3 biological replicates with 2 technical replicates each. Throughout, **** corresponds to P≤0.0001, *** indicates P ≤ 0.001, ** means P ≤ 0.01, * indicates ≤ 0.05, anything P > 0.05 is ns.

To evaluate whether bacterial internalization is necessary for robust immune activation, experiments were performed to compare immune responses in cells infected with NTHi strains with higher and lower internalization rates (**Figure 3**). Internalization rates were previously determined in PBECs in submerged serum-free culture (**Supplemental Figure 1C**). PBECs and NECs from 3 healthy control donors were grown in submerged culture and infected with highly internalized NTHi strain 398 (1.1% internalization) and less internalized strain 1607 (0.19% internalization), and incubated for up to 168 hours (7 days) (3A). NTHi significantly increased concentrations of IL-8 (CXCL8) in PBEC supernatants by 24 h post-infection, (2-way ANOVA with *post hoc* Sidaks)(3A). However, there was not a significant difference between strains at any timepoint. Similarly, IL-8 production in NECs is significantly elevated by exposure to NTHi by 24 hpi (p<0.001 by 2-way ANOVA) (3B). Analysis by qPCR showed that immune response to highly internalized NTHi strain 398 (1.1% internalization) was comparable with that of non-invasive lab strain 11931 (0% internalization) in Calu-3 cells exposed to bacteria for 24 hours (**Figures 3C–G**). Expression of pro-inflammatory markers IL-1β, CXCL8, CCL2, IL-6 and TNF was significantly upregulated in response to NTHi when evaluated using qPCR. However, there was not a substantial difference between gene expression in response to NTHi strain 398 compared with 11931 for any target (**Figure 3C–G**). We verified that this is not a strain-specific effect by using Cytochalasin D treatment to block internalization of NTHi strain 398 (Virji et al., 1992), demonstrating that reduction in internalized bacteria does not affect immune induction, except for IL1B (p=0.04 by linear test for trend over increasing cytochalasin D concentration), likely due to its activation via intracellular inflammasome sensing (**Supplemental Figure 3**). These data suggest that, although NTHi exposure is a potent activator of cytokines, invasion does not correlate with increased immune activation of pro-inflammatory cytokines. In models of lower airway epithelium, internalization does not appear to be critical for induction of CXCL8, IL6, CCL2 or TNF. This suggests that induction of these cytokines is driven primarily by NTHi components interacting with receptors on the cell surface, such as TLR2 and 4. However, there is a trend towards reduced induction of IL1B expression in cells which have been treated with cytochalasin D to block bacterial internalization (**Supplemental Figure 3**), suggesting that intracellular bacteria are necessary for full activation via intracellular inflammasome-associated NLRs. These data suggest that extracellular sensing of NTHi, perhaps by TLRs, dominate the induction of cytokines in the epithelium. Alternatively, there may be a threshold effect of intracellular bacterial sensing, so a dose-response may not be seen in response to additional bacteria when a minimum number of bacteria have already been internalized.

NTHi is a common commensal organism colonizing the nasopharynx without causing disease (Pichichero and Almudevar, 2016). However, infection by NTHi in the lung is capable of causing exacerbations in asthma and COPD patients and contributing to the progression of disease (Finney et al., 2014; Taylor et al., 2019). The mechanisms underlying the differential response of upper and lower airways remain poorly understood. Initial experiments evaluated differences in physical interactions between NTHi and the lower and upper respiratory tract epithelium, and demonstrated that in early infection, higher numbers of bacteria were internalized in upper airway compared with lower airway epithelium in primary cells cultured at ALI (significance by 2-way ANOVA p<0.0001) (**Supplemental Figure 7**). This suggests that the differential immune response leading to commensal colonization of the nasopharynx is not due to lower bacterial internalization or adherence.

**Figure 4:**
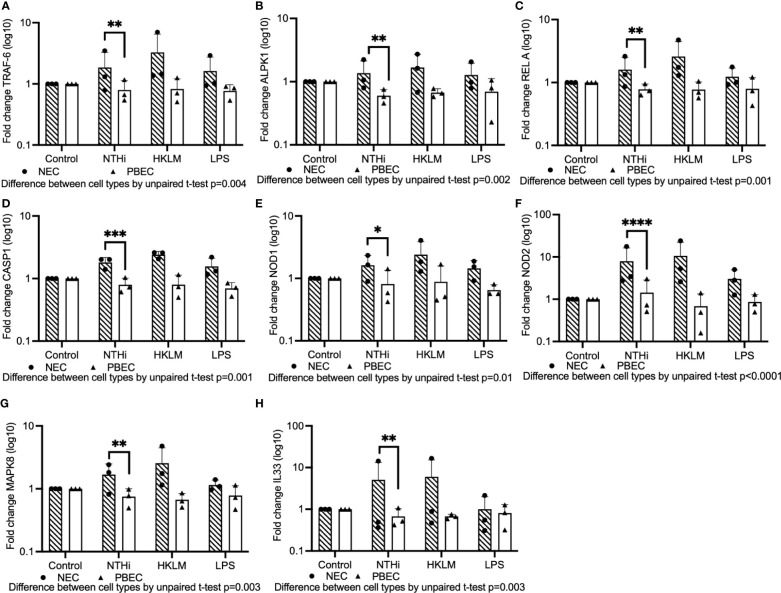
A Fluidigm Biomark PCR array experiment was performed to evaluate gene expression induced by NTHi in PBECs compared with NECs cultured at ALI. Mature ALI cultures were inoculated with a selection of NTHi strains, HKLM or LPS, and incubated in the presence of extracellular bacteria for 24 hours prior to isolation of RNA. **(A)** TLR-signaling pathway component TRAF6 was expressed at higher levels in NECs compared to PBECs inoculated with NTHi. **(B)** ALPK1 is more highly induced in NECs compared to PBECs. **(C)** Expression of NF-kB component RelA was significantly higher in NECs than PBECs stimulated with NTHi. **(D)** CASP1 was more highly activated in NECs than PBECs. **(E, F)** NOD1 and NOD2 were both expressed in NECs at significantly higher levels compared with PBECs exposed to NTHi. **(G)** MAPK8 was elevated in NECs compared with PBECs. **(H)** IL-33 was likewise elevated in NECs compared with PBECs. Significance for difference between infected NECs and PBECs for all results was evaluated using paired t-tests performed on log-transformed data. Means are indicated on graphs, and dots represent means of technical triplicates for each biological replicate from healthy control individuals. Throughout, **** corresponds to P ≤ 0.0001, *** indicates P ≤ 0.001, ** means P ≤ 0.01, * indicates ≤ 0.05, anything P > 0.05 is ns.

Using Fluidigm Biomark PCR array experiments, we evaluated whether signaling induced by NTHi differed in upper airway compared to lower epithelium using primary bronchial and nasal epithelial cells cultured at ALI (**Figure 4**). We found that expression of TRAF6, a factor downstream from TLR2 and TLR4, was significantly higher in NECs compared with PBECs (mean fold-change 1.86 vs 0.79 respectively, unpaired t-test p=0.004) in response to NTHi stimulation (**Figure 4A**). In addition, expression of downstream MYD88 and IRAK1 was numerically higher in NECs compared with PBECs, although this difference was not statistically significant (**Supplemental Figures 4A, B**).

Treatments with TLR2 and TLR4 agonists HKLM (Heat-killed *Listeria monocytogenes*) and LPS (Lipopolysaccharide) were included to evaluate differential TLR signaling in NECs compared with PBECs, but NECs responded to stimulation with HKLM and LPS at comparable or higher levels than PBECs, suggesting that they are capable of responding to TLR agonists (**Figure 4**).

Expression of ALPK1 (Alpha-protein kinase-1), which activates NF-kB via TRAF6 (Milivojevic et al., 2017; Cui et al., 2021), was also significantly higher in NECs compared with PBECs exposed to NTHi (mean fold-change 1.4 vs 0.6, unpaired t-test p=0.002)(**Figure 4B**). Evaluation of NF-kB subunit protein, RelA, which is involved in the translocation of NF-kB into the nucleus, demonstrated that expression was significantly higher in NECs compared with PBECs (mean fold-change 1.6 vs 0.8, unpaired t-test p=0.001)(**Figure 4C**).

Activation of the NLRP1 inflammasome was then evaluated. A downstream factor from NLR-associated inflammasomes, Caspase-1 (CASP1), was significantly more highly expressed in NECs compared with PBECs in cells stimulated with NTHi (mean fold-change 1.8 vs 0.8, unpaired t-test p=0.001) (**Figure 4D**). Evaluating another branch of the NOD signaling pathway, intracellular receptors NOD1 and NOD2 were both significantly more highly expressed in NECs than PBECs when exposed to NTHi (mean fold change 1.6 vs 0.8, unpaired t-test p=0.01 and 7.9 vs 1.4, unpaired t-test p<0.0001, respectively) (**Figures 4E, F**). MAPK8 (JNK1) expression, which is downstream from both TLR signaling via MYD88 (Arora et al., 2019), and NOD receptor signaling, was modestly induced in response to NTHi, and was significantly higher in NECs compared with PBECs (1.7 vs 0.8 mean fold-change, p=0.003 by unpaired t-test) (**Figure 4G**). Stimulation with NTHi only modestly induced MAPK3 (ERK1) in NECs, but overall expression was significantly higher than in PBECs by 2-way ANOVA (p=0.01) (**Supplemental Figure 4C**).

Expression of downstream pro-inflammatory cytokines was then evaluated. The alarmin IL-33 (IL33), which is induced in airway epithelial cells by a variety of infectious agents and allergic stimuli (Holgate et al., 2015), notably house dust mite allergen, was substantially induced by NTHi in NECs (mean fold-change 5.1), but unaffected in PBECs (difference between NECs and PBECs by unpaired t-test, p=0.003) (**Figure 4H**).

Similarly, expression of IL-8 (CXCL8) was modestly upregulated in NECs in response to NTHi and overall expression was significantly higher than in PBECs (2-way ANOVA NECs vs PBECs, p=0.007), whilst IL-1β (IL1B), a cytokine driven by inflammasome activation (Chen et al., 2018), was substantially induced in NECs and only slightly induced in PBECs, although this difference did not reach statistical significance (**Supplemental Figures 5A, B**). Both NECs and PBECs showed moderately upregulated IL-6 and TNF expression in response to NTHi (**Supplemental Figures 5C, D**).

Intercellular Adhesion Molecule 1 (ICAM-1) is a cell surface molecule used as a receptor by NTHi and major type rhinoviruses, and which may be upregulated by NTHi (Avadhanula et al., 2006; Sajjan et al., 2006). In this ALI co-culture model, ICAM-1 expression was moderately increased in NECs in response to NTHi, and overall was significantly higher than in PBECs (p=0.05 by 2-way ANOVA) (**Supplemental Figure 6A**). Evaluation of antimicrobial peptides showed that expression of lysozyme was not significantly upregulated in either cell type, whilst cathelicidin (CAMP) was modestly induced by NTHi at comparable levels in both NECs and PBECs (**Supplemental Figures 6B, C**).

Overall, these results suggest that immune induction by NTHi occurs more robustly in NECs compared with PBECs at 24 hpi. Due to the more pro-inflammatory nature of NTHi infections in the lower airways compared with the nasopharynx *in vivo* (Clementi and Murphy, 2011), these findings were unexpected. Different immune cell populations are present in the lower and upper airways, perhaps contributing differently to propagation of signaling from the epithelium, with a greater number of macrophages in the lower airways likely leading to greater amplification of immune signaling.

## Discussion

Our studies *in vitro* have demonstrated that NTHi can invade basal, secretory and ciliated epithelial cells of the human airway, and can drive inflammatory cytokine signaling in the absence of accessory inflammatory cells. Furthermore, whilst strains differ in their invasiveness, none appear to persist in cultured PBECs, and the induction of the inflammatory responses appears largely independent of the intracellular invasion.

Whilst biologic therapies targeting the type-2 cytokines IL-4, -5, -13 or epithelial alarmins are proving transformative in the management of severe type-2 high asthma (Holguin et al., 2020), for those with type-2 low inflammation, treatment options remain limited and pathology poorly understood (Hinks et al, 2021). Sputum neutrophilia in asthma is strongly associated with increased production of several cytokines including IL-1β, IL-6, CXCL8, IL-12, IL-17A and TNF (Sajjan et al., 2006; Yang et al., 2018; Hynes and Hinks, 2020), and this correlates with bacterial burden, in particular Gamma-proteobacteria (Brown et al., 2022). It is not clear if the source of these cytokines is predominantly inflammatory cells, including macrophages and neutrophils, or the airway epithelial cells which throughout the airways constitute the interface between host and the external world and the primary barrier against pathogens. We have found that NTHi potently induces primary human airway epithelial cells to express CXCL8, IL-1β, IL-6 and TNF, consistent with previous *in vitro* findings using cell lines (Sajjan et al., 2006), and therefore implicates NTHi as a significant driver of neutrophilic asthma. CXCL8, acting via its receptor CXCR2, is a major chemokine for neutrophils, whilst IL-6 activates JAK/STAT3 signaling pathways on neutrophils, macrophage and T cells expressing the IL-6R. IL-6 can also induce neutrophils to release soluble IL-6R which can act locally within the airway to activate eosinophils and act on airway epithelial cells to impair epithelial tight junction integrity and induce matrix metalloproteinases and the alarmin IL-33, contributing to pathology in asthma (Jevnikar et al., 2019). This IL-6 trans-signaling has similarly been observed recently in chronic obstructive pulmonary disease (COPD) in a subset of patients colonized with increased proteobacteria, particularly *Haemophilus* genus, where sIL-6R is correlated with markers of NETosis (Winslow et al., 2021).

Very few bacteria are capable of establishing long-term colonization of the lower airways, and those which do have necessarily evolved a variety of ways to evade clearance from the airways. *Haemophilus* species are primarily extracellular bacteria, however in clinical practice infections can be chronic and frequently recur following treatment with extracellularly-acting antibiotics such as beta-lactams (Van Schilfgaarde et al., 1999; Morey et al., 2011). Across all clinical-isolates tested we consistently observed rapid internalization of NTHi, albeit in low numbers. Contrary to expectation, once internalized, we did not detect NTHi persistence for more than 24 h, consistent with results seen in similar *in vitro* studies demonstrating that NTHi are viable but exist in a non-replicative state in airway epithelial cells (Morey et al., 2011; Baddal et al., 2015). It is nonetheless likely that this facultative intracellular lifestyle is important for NTHi capability to establish a niche within the airways for several reasons. Firstly, intracellular survival may be prolonged *in vivo*, where epithelial cell states may differ, but also where properties of the physical environment are different, notably significantly lower oxygen tensions. Hypoxia can occur in distal airways in the context of the mucus plugging which characterizes small airways disease in these patients (Montgomery et al., 2017; Dunican et al., 2021), and this could evoke metabolic changes in the *Haemophilus* to favor dormancy as occurs with other intracellular bacteria. Secondly, we observed over the course of 7 days that adherent NTHi could continue to enter the epithelium at a constant rate in our model system, so it is possible the capability to invade could provide temporary sanctuary from peak doses of extracellular antibiotics, where intermittent dosing or low bioavailability within the airway mucus layer mean antibiotics are at times subtherapeutic. Thirdly, whilst we showed equal propensity to invade three major classes of epithelial cell (ciliated, basal and secretory) which are detectable during ALI culture, it is plausible that the same common invasion mechanism allows them to enter and persist for longer in other more minor cell populations, such as ionocytes or in cells which have been altered by the type 2 inflammatory milieu. Fourthly, the apparent lack of persistent viable intracellular bacilli might be related to difficulty recovering viable bacteria using detergents or due to transition to a metabolic state which renders them unculturable, as occurs with other intracellular organisms such as *Campylobacter jejuni* (Watson and Galán, 2008). Besides these possibilities, NTHi may evade immune clearance via other additional mechanisms. NTHi is phagocytosed by airway macrophages, and has been observed within bronchoalveolar macrophages in asthma using FISH (Ackland et al., 2022), whilst in monocyte derived macrophages phagocytosed NTHi remains stably viable at 24 h post infection (Ackland et al., 2021). Furthermore, although NTHi does not produce extracellular polysaccharides required for classic biofilm formation (Moxon et al., 2008), it is protein and DNA rich (Gunn et al, 2016), and may persist extracellularly within biofilm-like structures formed from neutrophil breakdown products (Langereis and Hermans, 2013). Residence within biofilm-like structures may protect bacilli from antibiotics, extracellular killing and phagocytosis, and allow bacteria to provide ongoing epithelial cell stimulation and invasion. Indeed, neutrophil extracellular trap (NET) formation has been identified as a key marker of disease severity and responsiveness to azithromycin treatment in patients with bronchiectasis (Keir et al., 2021).

Based on our PCR data and the literature, we postulate that inflammatory signaling in response to NTHi may occur via three routes: TLR2 and 4, NOD1 and 2, and inflammasome-associated NLRs (NLRP1, NLRP3, NLRC4, AIM2). The first route is via TLR2 and 4 signaling, downstream IRF5 and 7, and via the MYD88-IRAK1-TRAF6 signaling transduction pathway (Jefferies, 2019). Another is via NOD1 and NOD2 sensing and downstream activation of MAPK signaling and NF-kB (Lee et al., 2019). Both TLR and NOD signaling appears to be more robustly induced in NECs compared with PBECs, but comparison of a larger number of donors is necessary to draw firm conclusions. Inflammasome-associated NLRs have also previously been implicated in NTHi-induced inflammatory signaling in a caspase-1-dependent manner resulting in cleavage of pro-IL-1β to its active form (Rotta detto Loria et al., 2013; Man et al., 2016; Chen et al., 2018). We have demonstrated equivalent levels of induction via NLRs in both NECs and PBECs using this model. NF-kB signaling is downstream from several of these mechanisms, in addition to possible activation via ALPK1. Infection with NTHi is also associated with transcriptional upregulation of cytokines CCL2 (MCP1), TNF, IL-1β, IL-6, CXCL8 and alarmins (TSLP, IL-33 and IL-25) (Khair et al, 1996; Patel et al., 2002; Sajjan et al., 2006; Meunier et al., 2015). Whilst induction of most cytokines was higher in NECs, CCL2 (MCP1) was also induced in PBECs at equivalent levels. CCL2 is a chemokine that mediates migration and infiltration of monocytes and macrophages, suggesting that immune signaling in the lower airways in response to NTHi may rely on recruiting immune cells to a greater extent than in the nasopharynx.

We observed similar induction of the proinflammatory mediators CXCL8, CCL2 and TNF in both highly invasive and less invasive strains, and in the presence or absence of the inhibitor of endocytosis cytochalasin D, suggesting that invasion was not required for innate activation of epithelial cells. Therefore, induction of these cytokines may be driven primarily by NTHi components interacting with receptors on the cell surface, such as TLR2 and 4. Alternatively, it possible that bacteria adherent to the epithelium could activate intracellular receptors without the requirement for invasion. Recent murine data show that extracellular gastrointestinal commensal microbes release membrane vesicles capable of delivering bacterial DNA to host cells to trigger tonic activation of the cytosolic cGAS-STING-IFN-I pathway which can occur in the absence of TLR4 recognition of bacterial LPS (Erttmann et al., 2022). Further studies will be needed to determine if NTHi can produce membrane vesicles capable of activating the NLRP3 inflammasome. In the case of IL-1β, inhibition by cytochalasin D did occur implying that full activation of the NLRP3 inflammasome requires bacterial internalization.

Contrary to our expectations, and consistently across a range of different NTHi strains, we observed stronger immune activation in nasal epithelial cells than in bronchial epithelial cells. How might this be explained, given an expectation that the nasal epithelium might tolerate NTHi more comfortably without inducing inflammation? It is possible this is an artefact of the model system and that the resistance to paracellular and intracellular invasion differs between cells grown *in vitro* and cells found *in situ* in their natural mucosal environment. Indeed, ALI culture is a highly reductionist and artificial system, although we have shown in PBECs that inflammatory response was not dependent on the degree of bacterial internalization. Alternatively, the interaction of NTHi with the nasal epithelium may be modulated significantly *in vivo* by the presence of other species in what is a much more complex, and high biomass, microbiome in upper than lower airway. Indeed, it has been recently reported that the common oral commensal *Rothia mucilaginosa* can inhibit innate inflammatory responses to *Pseudomonas aeruginosa, Staphylococcus aureus* or LPS in the A549 human pulmonary cell line *in vitro* (Rigauts et al., 2022). *R. mucilaginosa* also inhibited MIP-2 and other inflammatory cytokines after intranasal LPS challenge in mice. These effects seem to be mediated by cell-free supernatant and involve inhibition of NF-κB signaling upstream or at the level of the IκBα kinase complex.

Conversely, it is possible variations in the immune cell populations in the different tissues contribute to the tolerance of NTHi in the nasopharynx. In the nose and upper airways, dendritic cells form a tight network, draining pathogens to the lymph nodes to generate an adaptive immune response, whilst alveolar macrophages are the dominant immune cell type in the lower airways (Nicod, 2005). Dendritic cells are more efficient antigen-presenting cells, whilst alveolar macrophages are more effective phagocytes and may constitute an important reservoir of persistent NTHi infection in the lower airways (Gordon and Read, 2002). In addition, alveolar macrophages are capable of recruiting a large influx of neutrophils to the lower airways in more severe infections by generating neutrophil-recruiting chemokines such as CXCL8 and leukotriene B_4_ (Nicod, 2005). An *in vitro* model of NTHi infection in alveolar macrophages from COPD patients or healthy controls demonstrated NTHi induced steroid-resistant production of CXCL8, suggesting NTHi-induced inflammatory signaling in macrophages may contribute to steroid insensitivity in COPD and asthma (Khalaf et al., 2017). These data suggest that the modest signaling seen in the epithelial cells of the lower airways may be amplified by immune cells such as alveolar macrophages and neutrophils which are present in greater numbers in the lower airways compared with the nasopharynx (Nicod, 2005). Such anatomical compartmentalization of the mucosal immune system, leading to different immune responses at different sites, is becoming increasingly apparent. For instance, recent murine work has demonstrated imprinting of different T cell responses induced to *Listeria monocytogenes* by oral inoculation compared with gastric inoculation (Barreto de Alburquerque et al., 2022). Oral inoculation primed effector T cells which disseminated to lymphoid organs, lung and oral mucosa but did not home to the gut, whilst T cells induced by gastric inoculation were gut homing. These differences were attributable to lower dendritic cell expression of retinoic acid-producing enzymes in mandibular than mesenteric lymph nodes. It is therefore likely that anatomically distinct dendritic cells could significantly modulate mucosal immune responses in a number of qualities. Further research comparing the mechanisms of differential immune responses to bacteria like NTHi between upper and lower airways is likely to be fruitful.

Our findings are limited by being restricted to *in vitro* studies which do not fully replicate the complex immune environment within an intact human. Furthermore with PBEC samples there was marked variation in responses between donors, which is a well-known challenge associated with primary cell work. Further experiments using samples from a greater number of donors would be beneficial to confirm that these results are relevant to the wider population.

There are clear clinical implications for our findings. Although NTHi is often considered rather innocuous, our data support the growing body of data that this pathogen has a relatively unique ability to persist within the airways and drive ongoing and recurrent neutrophilic inflammation in type-2 low asthma, and therefore is likely to merit more attention by clinicians. This is a common clinical scenario and indeed using Nanopore long-read sequencing we have recently identified NTHi in the lower airways in 35% of a pilot cohort of patients with severe asthma (Jabeen et al., 2022). Further larger cohorts are required to define the prevalence of this and other specific potentially pathogenic bacteria in well-defined cohorts with airways disease. This will help to more accurately target appropriate use of long-term azithromycin therapy which has been shown in the AZISAST (Brusselle et al., 2013) and AMAZES (Gibson et al., 2017) trials to be effective in reducing exacerbations in severe asthma, and to be linked to colonization with NTHi (Taylor et al., 2019; Taylor et al., 2020). The ability of high initial NTHi abundance to predict responsiveness to azithromycin treatment, coupled with the decline in NTHi levels, suggest that the primary mechanism by which azithromycin reduces exacerbations is by eliminating NTHi infection and the accompanying inflammation, making this an important treatable trait in severe asthma. However, important questions remain. It is not known what is the effect of NTHi – and other bacteria – on the function and activation of airway cells *in vivo*, and how this responds to treatment. This could be answered by detailed sequential bronchoscopy studies applying novel single cell approaches. Secondly, azithromycin cannot be tolerated by all patients, it carries a high risk of inducing drug resistance (Serisier, 2013), and may be less effective against other significant pathogens like *Moraxella catarrhalis* or *Streptoccous pneumoniae*. The efficacy of other long-term antibiotics for modulation of airways diseases is unproven, so will require large, prospective, randomized clinical trials, such as the ongoing BEAT-severe asthma doxycycline trial (ISRCTN57935812). Thirdly, more research is needed to pinpoint where NTHi establishes its niche, either in epithelial cells, macrophages, or NETs. If NETs constitute the dominant mechanism of immune evasion then there may be clinical efficacy from therapeutics targeting NETs, such as the orally acting dipeptidyl peptidase 1 inhibitor Brensocatib (Chalmers et al., 2020).

In summary, our data highlight the pathogenic potential of NTHi in driving neutrophilic airways inflammation, point to a potential mechanism of immune evasion, and raise intriguing questions about the compartmentalization of mucosal immunity within the human respiratory tract.

## Data availability statement

The original contributions presented in the study are included in the article/**Supplementary Material**. Further inquiries can be directed to the corresponding author.

## Ethics statement

The studies involving human participants were reviewed and approved by Oxford Research Ethics Committee B (18/SC/0361) Leicestershire, Nottinghamshire and Rutland Ethics Committee, UK (08/H0406/189). The patients/participants provided their written informed consent to participate in this study.

## Author contributions

MB, SM, IP, CA-C and TH contributed to conceptualization and design of the study. MB, SM, GD and TH contributed to acquisition of study data. KH contributed to the development of the flow cytometry panel. Data were analyzed by MB, SM, IP, CA-C and TH. MB, TH drafted the manuscript which was approved by all authors.

## Supplementary material

Supplementary Figure 1 NTHi is able to replicate in media with PBECs in submerged culture, and some strains have the ability to be continually internalized for up to 7 days. **(A)** A CFU assay demonstrated that NTHi strains 398 and 1607 proliferate in serum-free media in the presence of PBECs. **(B)** Live intracellular NTHi are detectable by CFU assay up to 7 days post-infection in PBECs when extracellular bacteria are present. Strain 398 invades more rapidly, but the difference in invasion rate is reduced over time. **(C)** Live intracellular bacteria expressed as percent internalization. n=3 biological replicates with PBECs from three healthy donors and technical triplicates. Means and ranges are indicated on plots, and dots represent the mean of technical replicates for each donor.

Supplementary Figure 2 Gating strategy for flow cytometry experiment to evaluate proportions of each epithelial cell type infected with NTHi. PBECs cultured at ALI were infected with COPD strain 398 NTHi stained with CellTrace FarRed (CTFR) for 1 hour, treated with gentamicin for 1 hour to eliminate extracellular bacteria, and then stained for ciliated (tubulin), basal (CD49f) and goblet cell (CD66c) markers. Cells were flow-sorted to determine proportion of each cell type infected by NTHi. This gating strategy allowed us to isolate infected (CTFR+) and uninfected cells of each cell type. Flow plot shown from one representative experiment.

Supplementary Figure 3 Cytochalasin D reduces internalization of NTHi, but this does not diminish immune response. In submerged Calu-3 cell cultures inoculated with NTHi strain 398, treatment with cytochalasin D at concentrations up to 5 μg/ml did not affect bacterial viability **(A)**. Cytochalasin D reduced internalization rate of 398 in Calu-3 cells in submerged culture in a dose-dependent manner. Internalization by NTHi strain 398 is significantly reduced in Calu-3 cells pre-treated with cytochalasin D in increasing concentrations, in terms of both total numbers of internalized bacteria **(B)** and as a percent of total bacteria **(C)**. Significance due to concentration was evaluated using a 2-way ANOVA. Reduction in NTHi strain 398 internalization in cells treated with cytochalasin D did not result in reduced activation of IL-8 (CXCL8), IL6 or TNF **(D–F)**. However, IL1B was reduced in a dose-dependent manner **(G)**.

Supplementary Figure 4 Expression of MYD88, IRAK1 and MAPK3 in NECs versus PBECs. PBECs and NECs from 3 healthy control donors were cultured at ALI for a minimum of 28 days prior to inoculation with a selection of NTHi strains for 24 hours. RNA was isolated from these cultures and analyzed using the Fluidigm DeltaGene PCR array assay. **(A, B)**. MYD88 and IRAK1, downstream elements of TLR2/4 signaling, were numerically elevated in NECs compared with PBECs, but this was not statistically significant. **(C)** MAPK3 was modestly elevated in response to NTHi, but overall expression was significantly higher in NECs compared with PBECs (p=0.01 by 2-way ANOVA).

Supplementary Figure 5 Cytokine expression in NECs compared with PBECs in response to NTHi. PBECs and NECs from 3 healthy control donors were cultured at ALI for a minimum of 28 days prior to inoculation with a selection of NTHi strains for 24 hours. RNA was isolated from these cultures and analyzed using the Fluidigm DeltaGene PCR array assay. **(A)** IL-8 (CXCL8) expression was significantly higher in NECs inoculated with NTHi than PBECs. **(B)** IL1B was numerically higher in NECs compared with PBECs. **(C, D)**. IL-6 and TNF were upregulated in response to NTHi in both NECs and PBECs. Difference between cell types was evaluated by 2-way ANOVA and *post-hoc* Sidaks multiple comparisons test. Means are indicated on graphs and dots represent the means of technical replicates for each donor.

Supplementary Figure 6 Surface molecule ICAM-1 and antimicrobial peptide expression in response to NTHi. PBECs and NECs from 3 healthy control donors were cultured at ALI for a minimum of 28 days prior to inoculation with NTHi strains for 24 hours. RNA was isolated from these cultures and analyzed using the Fluidigm DeltaGene PCR array assay. **(A)** ICAM-1 was increased in NECs compared with PBECs in infected cells. **(B, C)**. Lysozyme and CAMP were expressed at equal levels in both NECs and PBECs, with modest upregulation of CAMP seen in both cell types in response to NTHi. Difference between cell types was evaluated by 2-way ANOVA and *post-hoc* Sidaks multiple comparisons test. Means are indicated on graphs and dots represent the means of technical replicates for each donor.

Supplementary Figure 7 NTHi is internalized at higher rates in NECs compared with PBECs. PBECs cultured at ALI were infected with COPD strain 398 NTHi stained with CellTrace FarRed for 1 hour, treated with gentamicin for 1 hour to eliminate extracellular bacteria, and then stained for ciliated, basal and goblet cell markers. Cells were flow-sorted to determine proportion of each cell type infected by NTHi. In NECs, secretory cells were the most highly infected cell type, closely followed by ciliated cells, with basal cells being the least infected, in terms of both absolute numbers and percent **(A, B)**. **(A)** The total number of cells harboring intracellular NTHi was higher in NECs than in PBECs in secretory and ciliated cells, but there was no difference in basal cells. Overall, there was a significantly greater number of infected NECs compared with PBECs **(B)**. The percentage of infected cells was also higher in NECs than PBECs in all cell types. Difference between cell types was evaluated by 2-way ANOVA and *post-hoc* Sidaks multiple comparisons test. n=3 biological replicates from different healthy donors.

Supplementary Table 8 NTHi strain information and references Table listing strain information and relevant references in the literature. *sr indicates that the strain is streptomycin resistant.

Supplementary Table 9 qPCR primer list Table listing qPCR primer sequences used in this manuscript, with the exception of proprietary Fluidigm PCR array primers.

Supplementary Data Sheet 1 PCR array raw data Data files containing raw data for the Fluidigm Biomark PCR Array experiments performed on NECs and PBECs at ALI, as described in .
