# Supplementary figures and images for: Epithelial immune activation and intracellular invasion by non-typeable Haemophilus influenzae
Source: Front Cell Infect Microbiol. 2023 Apr 24;13:1141798. doi: 10.3389/fcimb.2023.1141798 (PMC10167379; doi:10.3389/fcimb.2023.1141798)

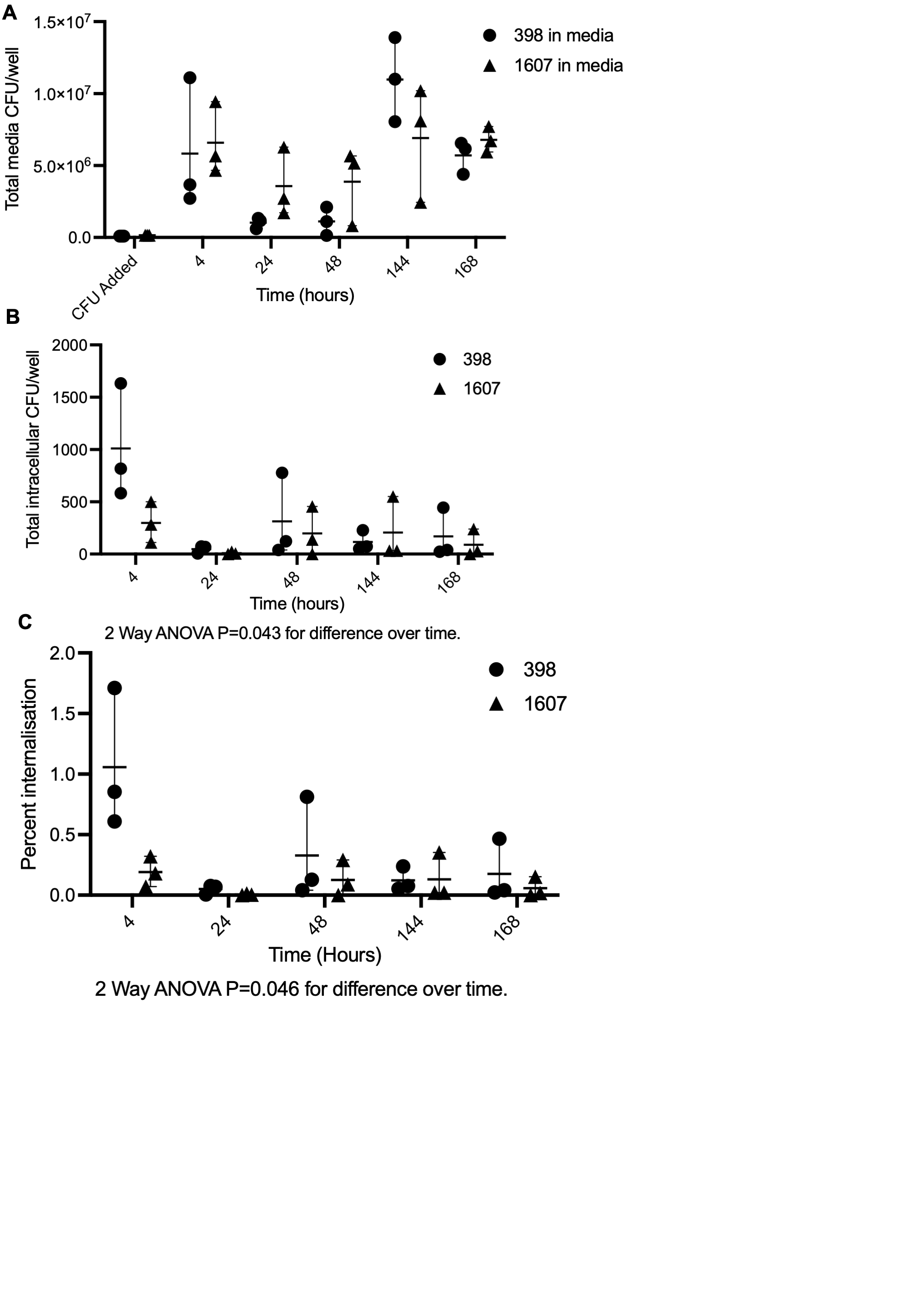

Supplement: Supplementary Figure 1 — NTHi is able to replicate in media with PBECs in submerged culture, and some strains have the ability to be continually internalized for up to 7 days. (A) A CFU assay demonstrated that NTHi strains 398 and 1607 proliferate in serum-free media in the presence of PBECs. (B) Live intracellular NTHi are detectable by CFU assay up to 7 days post-infection in PBECs when extracellular bacteria are present. Strain 398 invades more rapidly, but the difference in invasion rate is reduced over time. (C) Live intracellular bacteria expressed as percent internalization. n=3 biological replicates with PBECs from three healthy donors and technical triplicates. Means and ranges are indicated on plots, and dots represent the mean of technical replicates for each donor. [file Image_1.tiff]

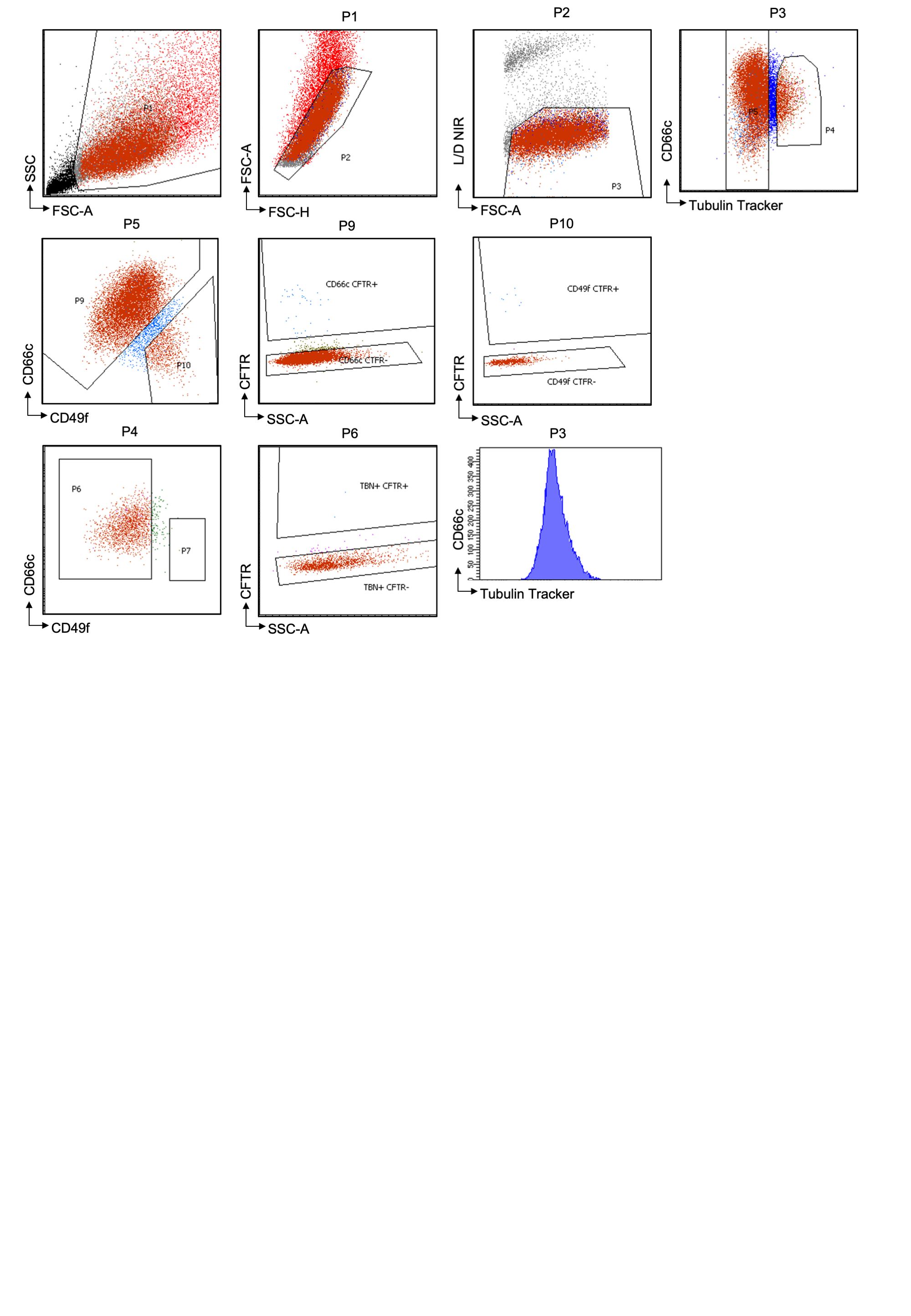

Supplement: Supplementary Figure 2 — Gating strategy for flow cytometry experiment to evaluate proportions of each epithelial cell type infected with NTHi. PBECs cultured at ALI were infected with COPD strain 398 NTHi stained with CellTrace FarRed (CTFR) for 1 hour, treated with gentamicin for 1 hour to eliminate extracellular bacteria, and then stained for ciliated (tubulin), basal (CD49f) and goblet cell (CD66c) markers. Cells were flow-sorted to determine proportion of each cell type infected by NTHi. This gating strategy allowed us to isolate infected (CTFR+) and uninfected cells of each cell type. Flow plot shown from one representative experiment. [file Image_2.tiff]

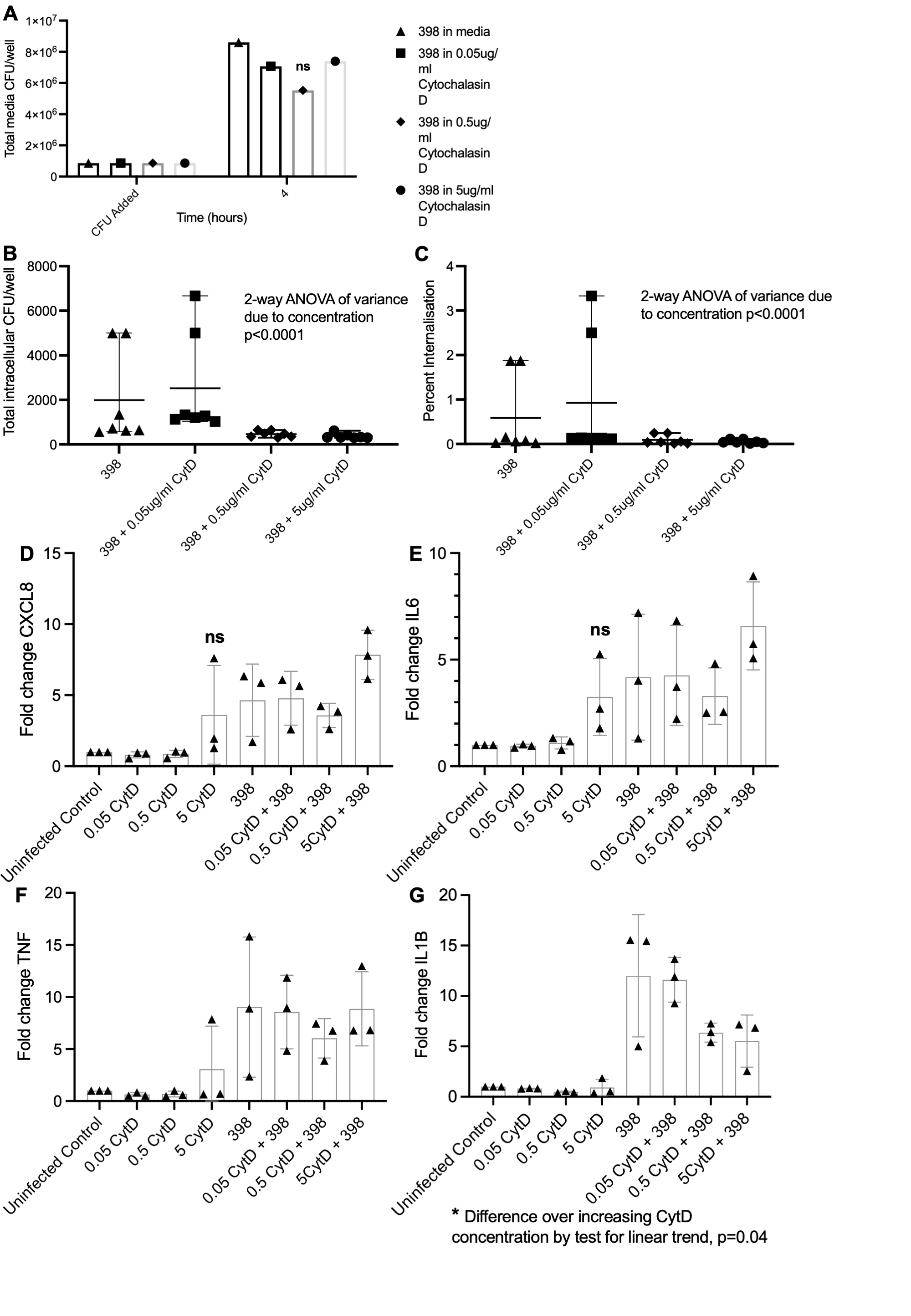

Supplement: Supplementary Figure 3 — Cytochalasin D reduces internalization of NTHi, but this does not diminish immune response. In submerged Calu-3 cell cultures inoculated with NTHi strain 398, treatment with cytochalasin D at concentrations up to 5 μg/ml did not affect bacterial viability (A). Cytochalasin D reduced internalization rate of 398 in Calu-3 cells in submerged culture in a dose-dependent manner. Internalization by NTHi strain 398 is significantly reduced in Calu-3 cells pre-treated with cytochalasin D in increasing concentrations, in terms of both total numbers of internalized bacteria (B) and as a percent of total bacteria (C). Significance due to concentration was evaluated using a 2-way ANOVA. Reduction in NTHi strain 398 internalization in cells treated with cytochalasin D did not result in reduced activation of IL-8 (CXCL8), IL6 or TNF (D–F). However, IL1B was reduced in a dose-dependent manner (G). [file Image_3.tiff]

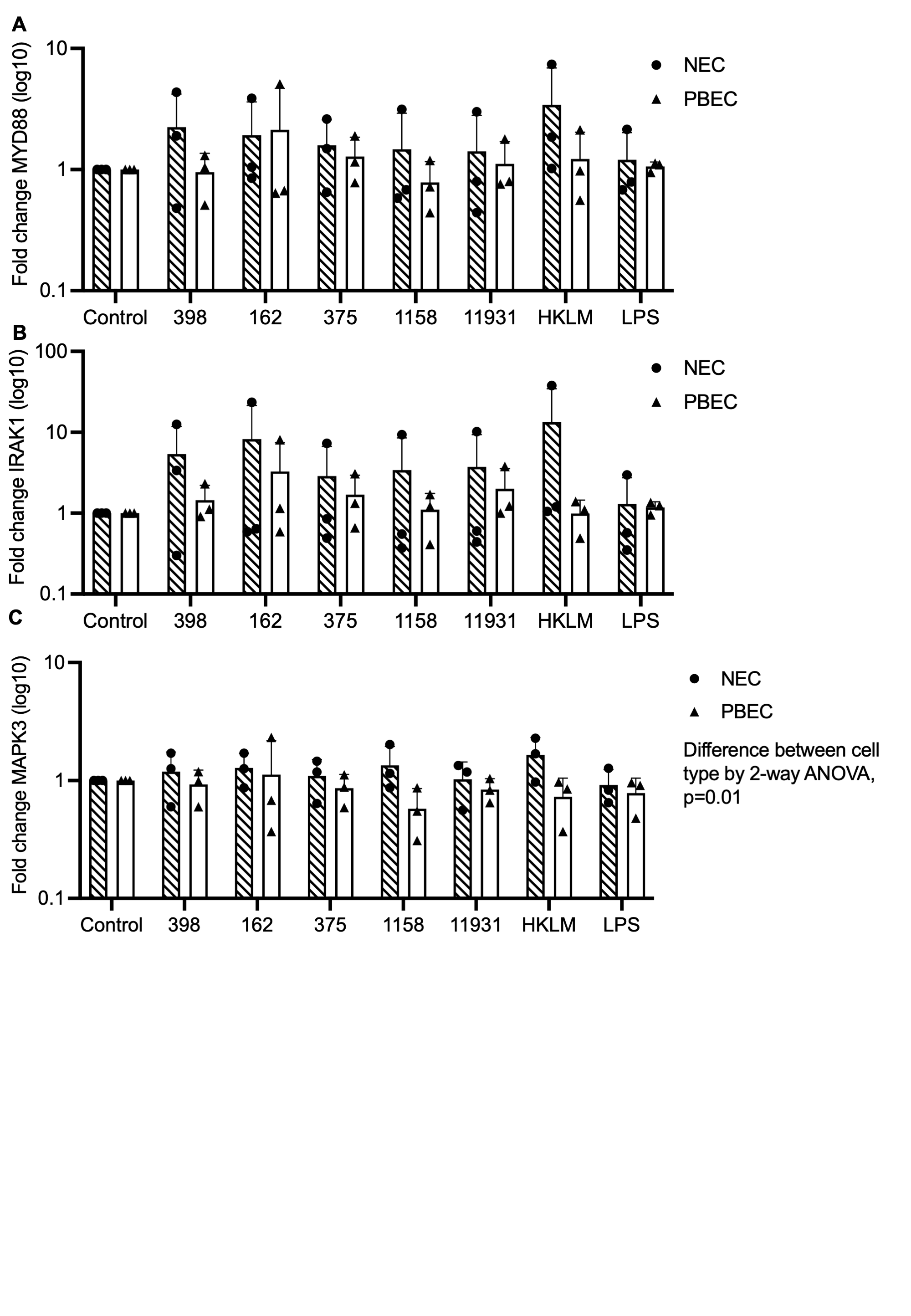

Supplement: Supplementary Figure 4 — Expression of MYD88, IRAK1 and MAPK3 in NECs versus PBECs. PBECs and NECs from 3 healthy control donors were cultured at ALI for a minimum of 28 days prior to inoculation with a selection of NTHi strains for 24 hours. RNA was isolated from these cultures and analyzed using the Fluidigm DeltaGene PCR array assay. (A, B). MYD88 and IRAK1, downstream elements of TLR2/4 signaling, were numerically elevated in NECs compared with PBECs, but this was not statistically significant. (C) MAPK3 was modestly elevated in response to NTHi, but overall expression was significantly higher in NECs compared with PBECs (p=0.01 by 2-way ANOVA). [file Image_4.tiff]

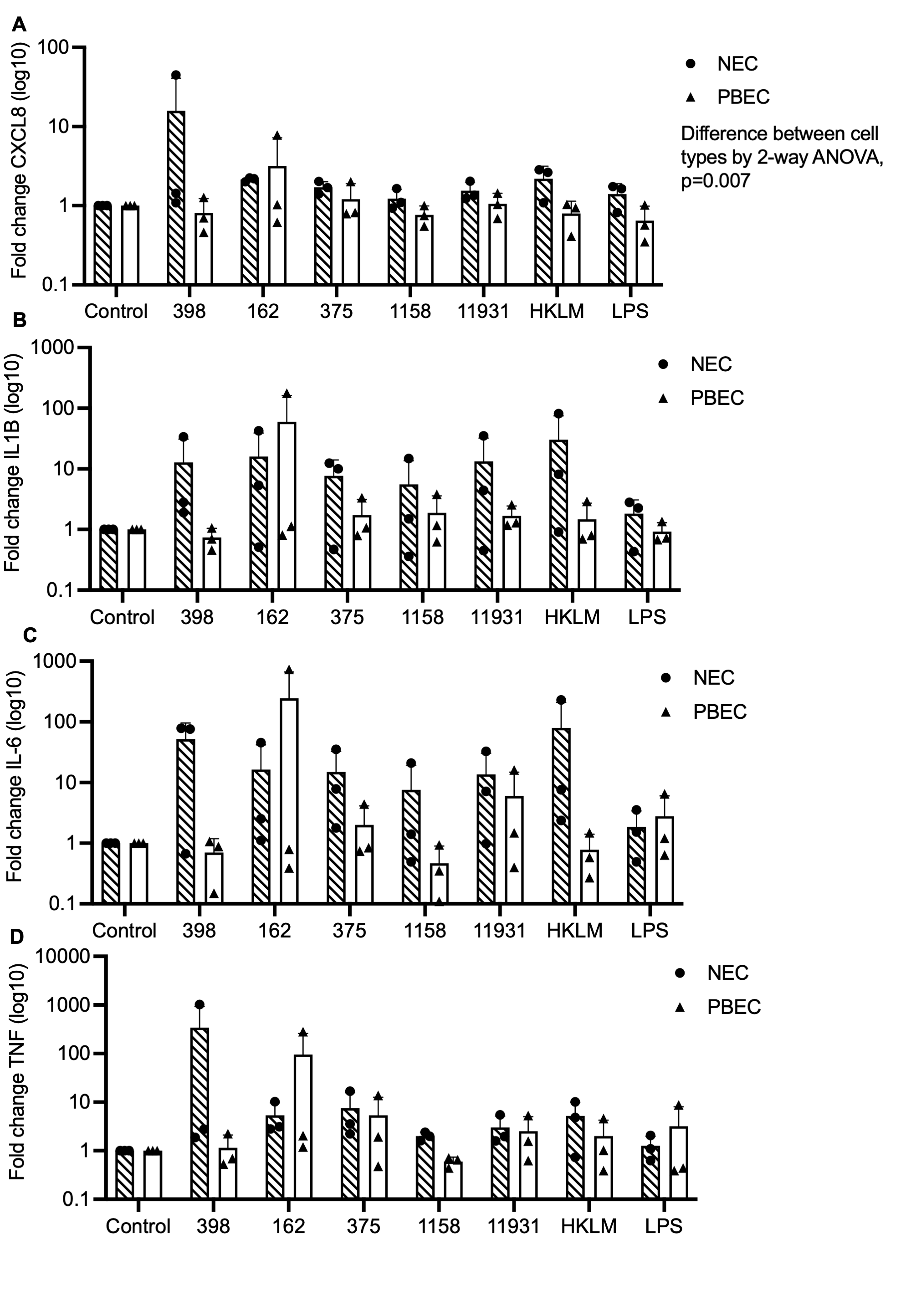

Supplement: Supplementary Figure 5 — Cytokine expression in NECs compared with PBECs in response to NTHi. PBECs and NECs from 3 healthy control donors were cultured at ALI for a minimum of 28 days prior to inoculation with a selection of NTHi strains for 24 hours. RNA was isolated from these cultures and analyzed using the Fluidigm DeltaGene PCR array assay. (A) IL-8 (CXCL8) expression was significantly higher in NECs inoculated with NTHi than PBECs. (B) IL1B was numerically higher in NECs compared with PBECs. (C, D). IL-6 and TNF were upregulated in response to NTHi in both NECs and PBECs. Difference between cell types was evaluated by 2-way ANOVA and post-hoc Sidaks multiple comparisons test. Means are indicated on graphs and dots represent the means of technical replicates for each donor. [file Image_5.tiff]

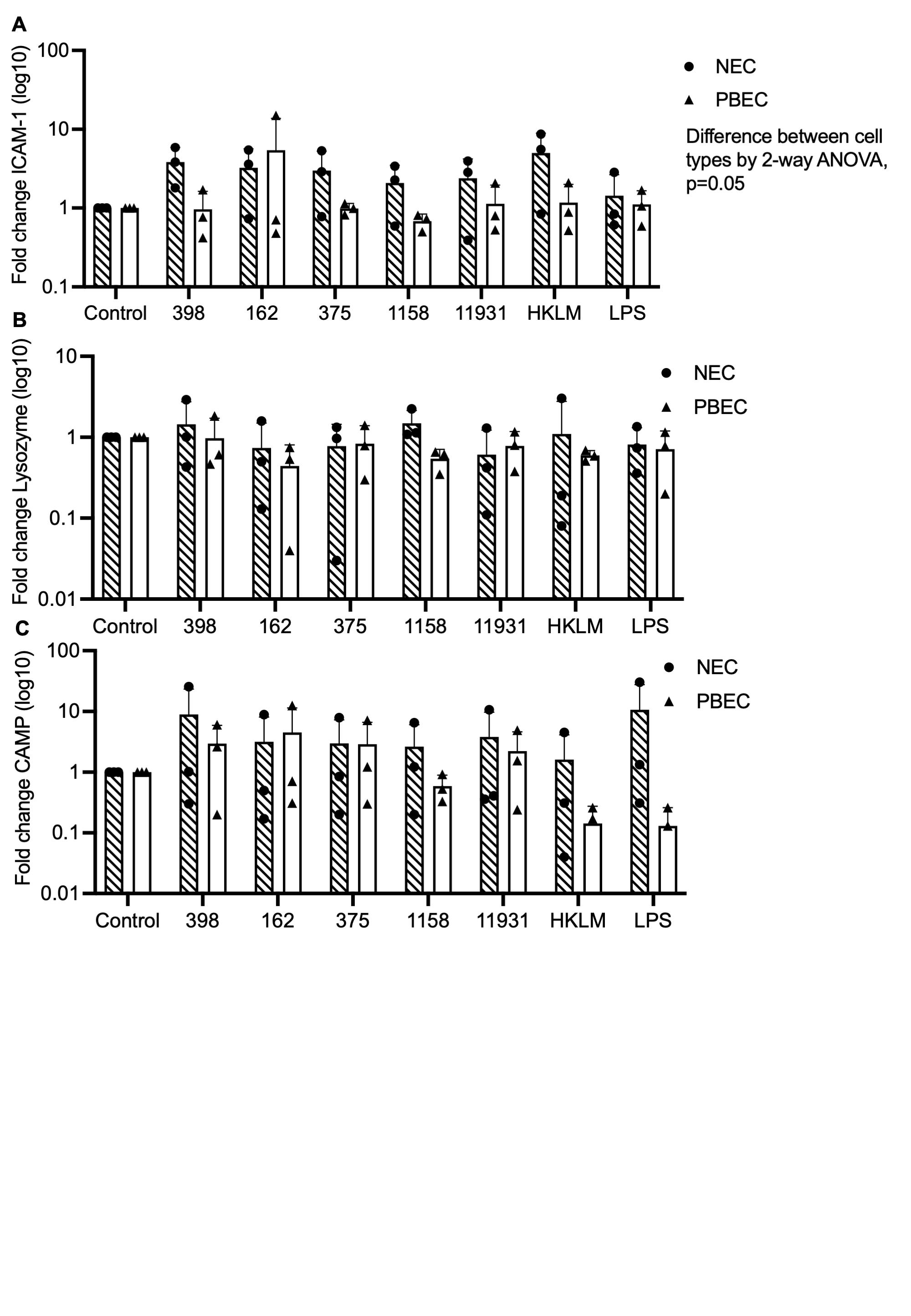

Supplement: Supplementary Figure 6 — Surface molecule ICAM-1 and antimicrobial peptide expression in response to NTHi. PBECs and NECs from 3 healthy control donors were cultured at ALI for a minimum of 28 days prior to inoculation with NTHi strains for 24 hours. RNA was isolated from these cultures and analyzed using the Fluidigm DeltaGene PCR array assay. (A) ICAM-1 was increased in NECs compared with PBECs in infected cells. (B, C). Lysozyme and CAMP were expressed at equal levels in both NECs and PBECs, with modest upregulation of CAMP seen in both cell types in response to NTHi. Difference between cell types was evaluated by 2-way ANOVA and post-hoc Sidaks multiple comparisons test. Means are indicated on graphs and dots represent the means of technical replicates for each donor. [file Image_6.tiff]

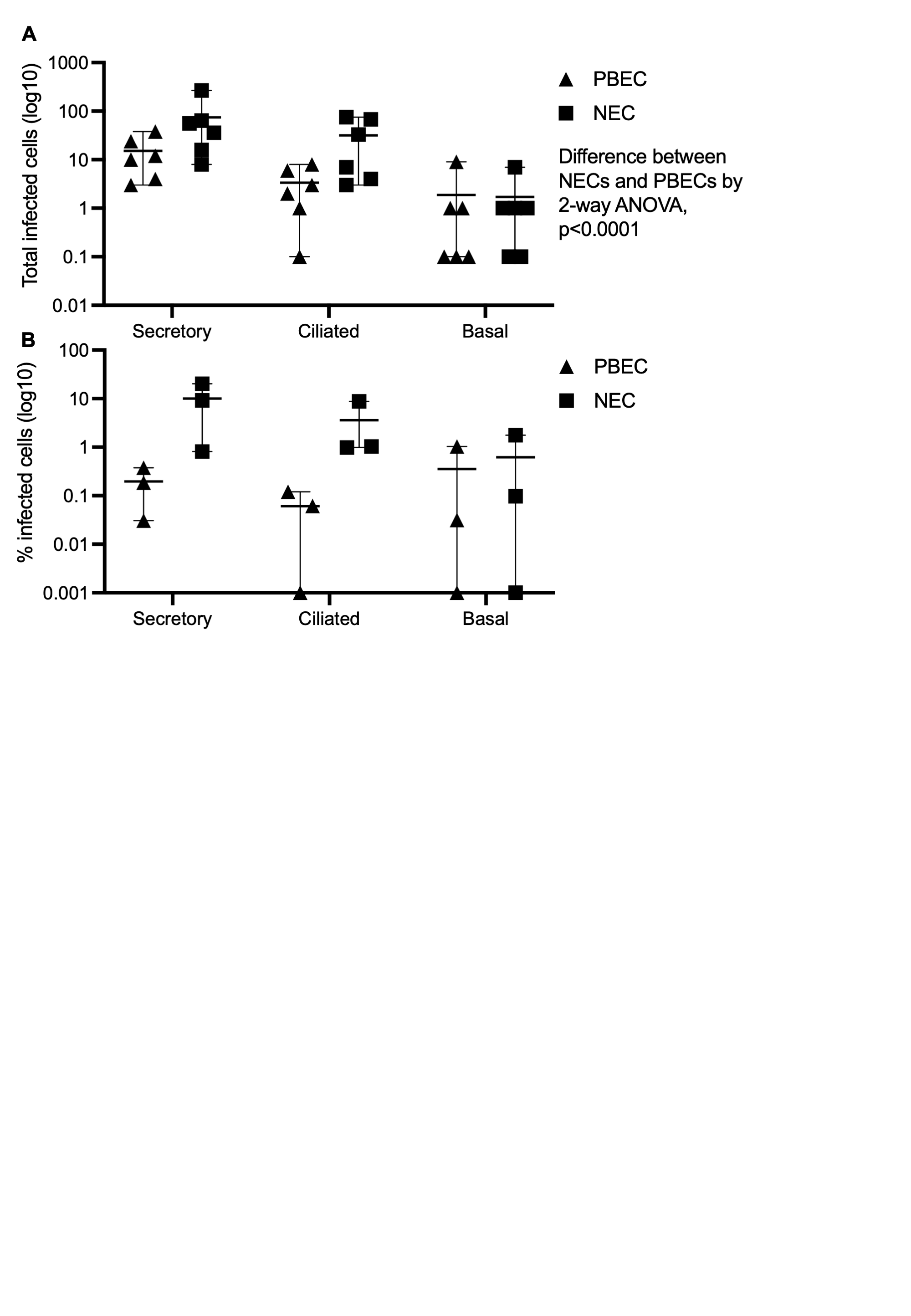

Supplement: Supplementary Figure 7 — NTHi is internalized at higher rates in NECs compared with PBECs. PBECs cultured at ALI were infected with COPD strain 398 NTHi stained with CellTrace FarRed for 1 hour, treated with gentamicin for 1 hour to eliminate extracellular bacteria, and then stained for ciliated, basal and goblet cell markers. Cells were flow-sorted to determine proportion of each cell type infected by NTHi. In NECs, secretory cells were the most highly infected cell type, closely followed by ciliated cells, with basal cells being the least infected, in terms of both absolute numbers and percent (A, B). (A) The total number of cells harboring intracellular NTHi was higher in NECs than in PBECs in secretory and ciliated cells, but there was no difference in basal cells. Overall, there was a significantly greater number of infected NECs compared with PBECs (B). The percentage of infected cells was also higher in NECs than PBECs in all cell types. Difference between cell types was evaluated by 2-way ANOVA and post-hoc Sidaks multiple comparisons test. n=3 biological replicates from different healthy donors. [file Image_7.tiff]

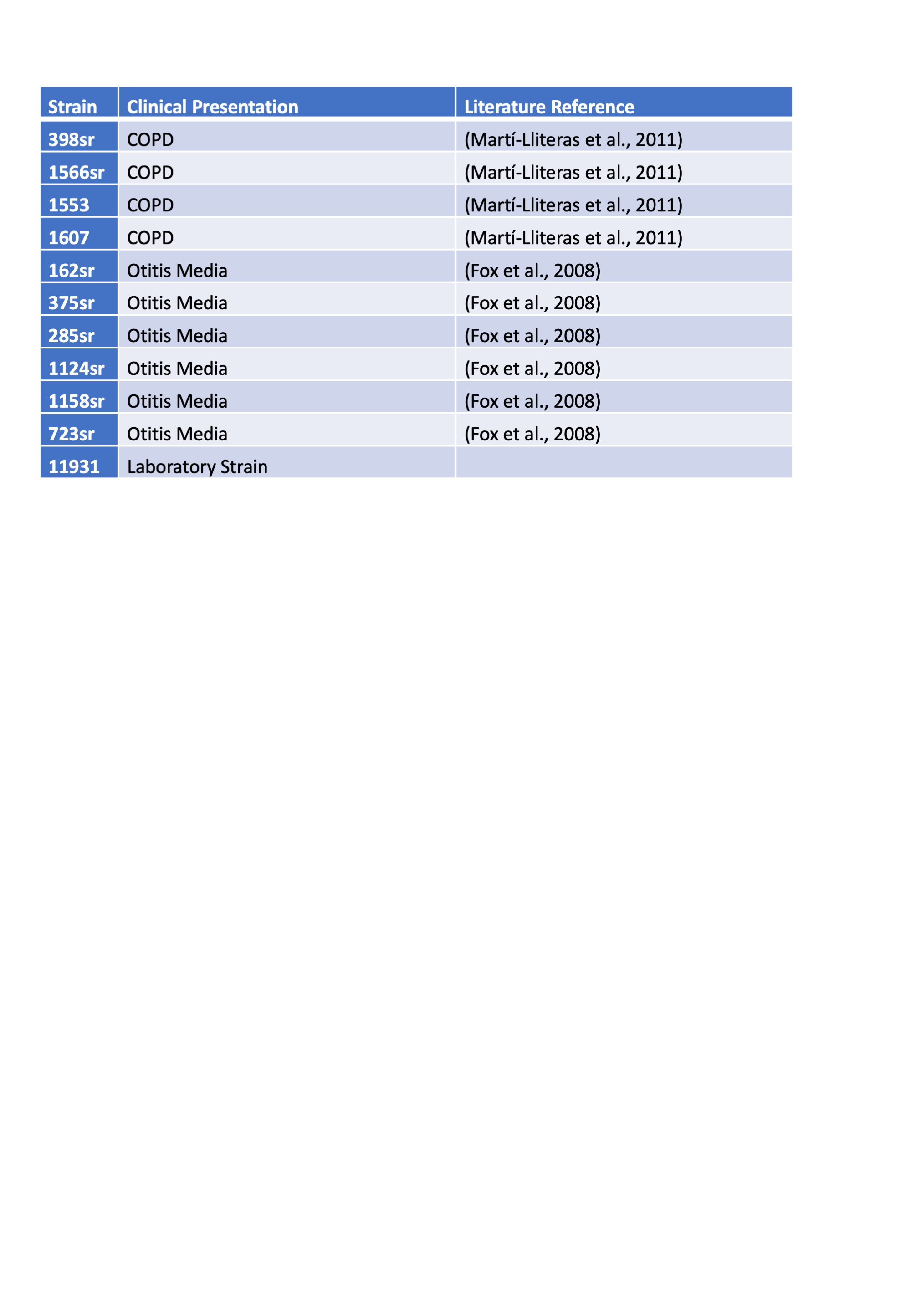

Supplement: Supplementary Table 8 — NTHi strain information and references Table listing strain information and relevant references in the literature. *sr indicates that the strain is streptomycin resistant. [file Image_8.tiff]

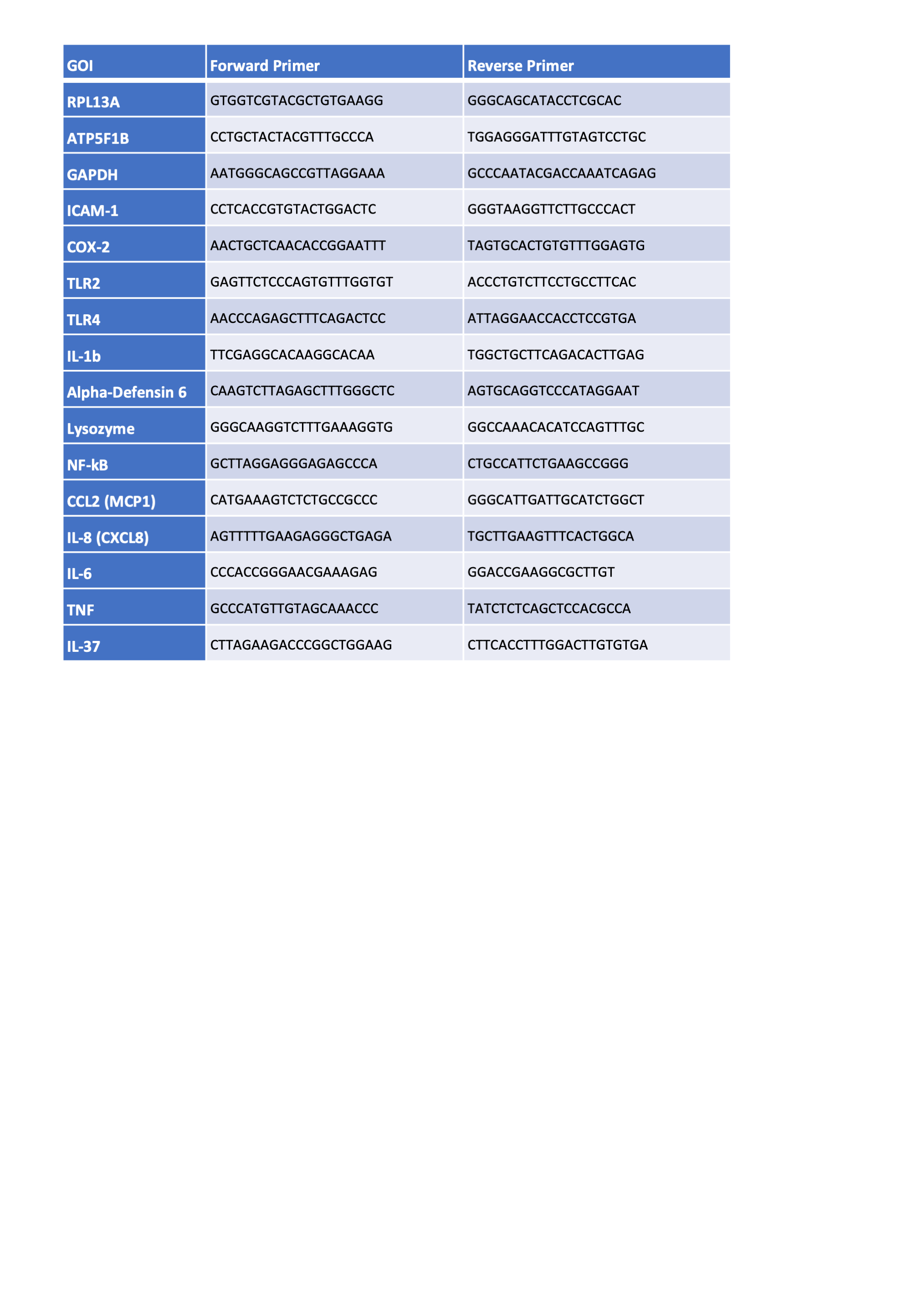

Supplement: Supplementary Table 9 — qPCR primer list Table listing qPCR primer sequences used in this manuscript, with the exception of proprietary Fluidigm PCR array primers. [file Image_9.tiff]
